# Pancreatoduodenectomy and surgical treatment of groove pancreatitis

**DOI:** 10.1590/0102-67202025000026e1895

**Published:** 2025-08-29

**Authors:** Franz Robert APODACA-TORREZ, Orlando Rondan ZOTTI, Marcio APODACA-RUEDA, Mariana Araújo SANTOS, Rogério Aoki FUZIY, Edson José LOBO

**Affiliations:** 1 Universidade Federal de São Paulo, Escola Paulista de Medicina, Surgical Gastroenterology Unit, Pancreatobiliary Division – São Paulo (SP), Brazil.

**Keywords:** Chronic Pancreatitis, Pancreaticoduodenectomy, Alcoholic Pancreatitis, Pancreatite Crônica, Pancreaticoduodenectomia, Pancreatite Alcoólica

## Abstract

**Background:**

Groove pancreatitis (GP) is a rare and segmental form of chronic pancreatitis that affects the pancreaticoduodenal sulcus. Its pathophysiology is still not well known, and several etiological factors have been attributed, with chronic alcohol consumption being the most common association. Its treatment still generates controversy. The initial clinical approach followed by endoscopic therapies prevails. Surgery is indicated when these treatment options fail.

**Aims:**

The aim of this study was to analyze the clinical, imaging, and surgical treatment data of a series of patients diagnosed with GP.

**Methods:**

The clinical, radiological, surgical, and postoperative follow-up data were analyzed, in addition to the histopathological results of chronic pancreatitis, in patients undergoing pancreaticoduodenectomy.

**Results:**

A total of eight patients were included, of whom six were male, and their mean age was 45 years. The main symptom presented was long-standing abdominal pain with the use of analgesics and weight loss; all patients were chronic alcoholics. Imaging methods defined the diagnosis of GP in the preoperative period in five patients. In three patients, the preoperative diagnosis was neoplasia of the head of the pancreas. All patients underwent pancreaticoduodenectomy and one patient developed pancreatic fistula. There was a regression of pain in all patients.

**Conclusions:**

For patients with GP who do not respond to the clinical approach, or in the face of diagnostic doubt, pancreaticoduodenectomy constitutes a good therapeutic option.

## ARTICLE HIGHLIGHTS

- Groove pancreatitis is an unusual form of chronic pancreatitis that can be mistaken for a pancreatic head neoplasm.
- Once the diagnosis is confirmed, clinical management follows the standard recommendations for chronic pancreatitis.
- Surgery is indicated when clinical treatment fails or when there is diagnostic uncertainty regarding pancreatic neoplasia.
- Pancreatoduodenectomy is an effective treatment option when performed in high-volume referral centers.

## CENTRAL MESSAGE

Groove pancreatitis (GP) is a rare, segmental form of chronic pancreatitis that primarily affects individuals between 40 and 50 years of age. It has been referred to by various other names, such as paraduodenal pancreatitis, cystic dystrophy of heterotopic pancreas, duodenal dystrophy, duodenal pancreatic hamartoma, paraduodenal wall cyst, and myoadenomatosis. This distinct and sporadic form of pancreatitis (GP) can be classified, depending on the affected segment, into a segmental form — affecting the entire pancreatic head — and a pure form limited to the pancreaticoduodenal groove, with preservation of the remaining pancreatic parenchyma. Its true incidence, as well as its pathophysiological mechanisms, remains unknown.

## PERSPECTIVES

Groove pancreatitis is a rare, segmental form of chronic pancreatitis that, in some cases, may be mistaken for pancreatic head neoplasia, with imaging modalities including endoscopic ultrasound currently playing a key role in its diagnosis. A review of the medical papers indicates that initial treatment should be multidisciplinary, similar to the management of classic chronic pancreatitis. When there is little or no response to conservative and/or endoscopic treatment, surgical intervention is indicated, and pancreatoduodenectomy is a good option, provided it is performed in centers with extensive experience in pancreatobiliary surgery.

## VISUAL ABSTRACT

**Figure:**
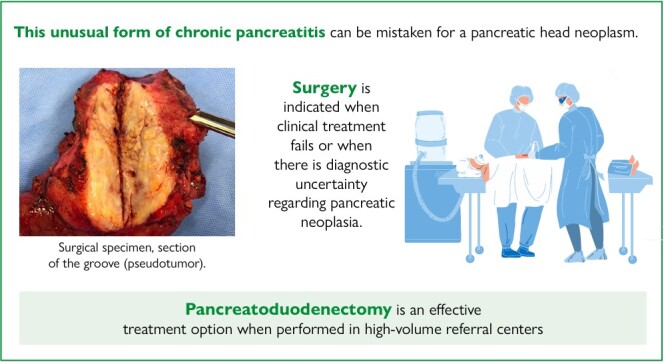

## RESUMO

**Racional:**

A pancreatite do sulco (PS) é uma forma rara e segmentar de pancreatite crônica que afeta o sulco pancreatoduodenal, cuja fisiopatologia ainda não é bem conhecida e se atribuem vários fatores etiológicos, sendo o consumo crônico do álcool a associação mais comum. O tratamento da mesma ainda gera controvérsias. Prevalece a abordagem clínica inicial seguida de terapêuticas endoscópicas. A cirurgia está indicada diante da falha das mesmas.

**Objetivos:**

Analisar os dados clínicos, de imagem e do tratamento cirúrgico de uma série de doentes com diagnóstico de pancreatite do sulco.

**Métodos:**

Foram analisados dados clínicos, radiológicos, da cirurgia e do acompanhamento pós-operatório, além do resultado histopatológico de pancreatite crônica de pacientes submetidos a pancreatoduodenectomia,

**Resultados:**

Foram incluídos oito doentes, seis do sexo masculino; a média de idade foi de 45 anos. O principal sintoma apresentado foi a dor abdominal de longa data de evolução em uso de analgésicos e a perda de peso; todos os pacientes eram etilistas crônicos. Os métodos de imagem definiram o diagnóstico de pancreatite do sulco ainda no pré-operatório em cinco doentes. Em três doentes, o diagnóstico pré-operatório foi de neoplasia de cabeça de pâncreas. Todos os doentes foram submetidos à pancreatoduodenectomia e um doente evoluiu com fístula pancreática. Houve regressão da dor em todos os pacientes.

**Conclusões:**

Pacientes com pancreatite do sulco que não respondem à abordagem clínica, ou, diante da dúvida diagnóstica, a pancreatoduodenectomia constitui-se em uma boa opção terapêutica.

## INTRODUCTION

 Groove pancreatitis (GP) is a rare, segmental form of chronic pancreatitis that affects the dorsal and superior regions of the pancreatic head (groove), located between the pancreas, bile duct, and duodenum. One of its distinguishing features is the preservation of the pancreatic parenchyma in the body and tail. It was first described by Becker in 1973 under the term *Rinnenpankreatitis* ^5^, and the term *groove pancreatitis* was introduced in 1982 by Stolte et al.^21^ This distinct and sporadic form of pancreatitis (GP) can be classified according to the affected region: the segmental form, which involves the entire pancreatic head, and the pure form, which is limited to the pancreaticoduodenal groove, preserving the remaining pancreatic parenchyma. Its pathophysiological aspects are not well understood, though mechanisms such as Kaiser’s phenomenon (parenchymal ischemia due to fibrosis and scarring), hypertrophy of Brunner’s glands in the duodenal wall^6^, ectopic pancreatic tissue in the duodenal wall, and obstruction of the minor duodenal papilla have been proposed. Multiple terms have been used in the medical publications to refer to this same condition^1^. 

 Etiological factors described include chronic alcoholic pancreatitis, smoking, pancreatic trauma, duodenal diverticulum, and anomalies of the duct of Santorini^14^. In many cases of GP, distinguishing it from pancreatic cancer becomes a diagnostic challenge^18^. 

 Once the diagnosis of GP is confirmed, there is no single standard treatment for this unique form of pancreatitis. Management may range from medical therapy with symptomatic treatment and alcohol abstinence to endoscopic or surgical approaches^8,13^. Surgical treatment is indicated when other modalities fail or when a neoplastic process cannot be completely ruled out, with pancreatoduodenectomy (PD) being one of the techniques reported to yield the best outcomes^13,19,20,25^ . 

 The objective of this study was to analyze the outcomes of a case series of patients with GP who underwent PD. 

## METHODS

 This is a retrospective cross-sectional study involving patients with a histopathologically confirmed diagnosis of segmental chronic pancreatitis (pure and segmental forms) who underwent PD between 2008 and 2023, performed by the Pancreatobiliary Group of the Division of Surgical Gastroenterology at Escola Paulista de Medicina – UNIFESP. The study protocol was submitted to and approved by the Research Ethics Committee of the Federal University of São Paulo under approval number 6.996.180. 

 Data were collected through the analysis of physical and electronic medical records. The following variables were recorded: age, sex, habits, clinical presentation, imaging findings, surgical procedures, complications, histopathological results, and postoperative clinical outcomes. Data were compiled and organized in an Excel spreadsheet (Microsoft 2017, USA). Quantitative variables were presented as mean and standard deviation (SD) and qualitative variables as frequency and percentage. 

## RESULTS

 A total of eight patients were identified, of whom six were male (75%). The patients’ mean age was 45 years (SD±6.69). The main symptoms were abdominal pain and weight loss (WL), present in seven patients (87.5%), along with symptoms of delayed gastric emptying (DGE), observed in four patients (50%). Cholestatic signs and symptoms were noted in two patients (25%). All patients had a history of chronic alcohol consumption and only one was not a heavy smoker (100% and 87.5%, respectively). 

**Figure 1:**
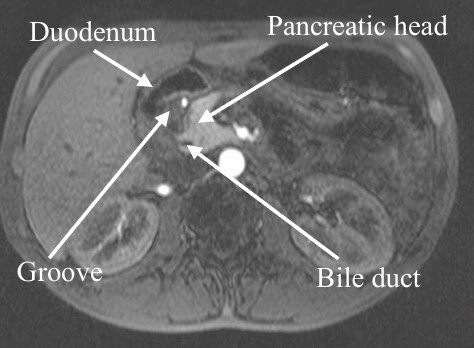
T2-weighted MRI of a patient with groove pancreatitis showing thickening of the groove, a pancreas with normal appearance, and a non-dilated common bile duct (arrows).

 Among the imaging methods performed, upper abdominal ultrasound was conducted in four patients; however, the results were inconclusive. Contrast-enhanced abdominal computed tomography (CT) was performed in all patients, which revealed significant findings, such as enlargement of the pancreatic head due to thickening of the pancreaticoduodenal groove, cysts in this region, and thickening of the second portion of the duodenal wall, suggesting GP in five patients. In three cases, a mass in the region of the pancreatic head was observed, suggestive of neoplasia, along with mild dilation of the main pancreatic duct in the body and tail. Magnetic resonance imaging (MRI) was performed in five patients, which demonstrated the same findings as CT, though it better characterized the cystic formations in the groove and thickening of the duodenal wall (Figure 1); in two patients, it also showed dilation of the biliary tract and the main pancreatic duct. Endoscopic ultrasound (EUS) was performed in six patients, which revealed findings suggestive of GP in four of them, including the presence of small anechoic cysts, thickening of the pancreaticoduodenal groove, hyperechoic striations, and thickening of the duodenal wall. 

**Figure 2:**
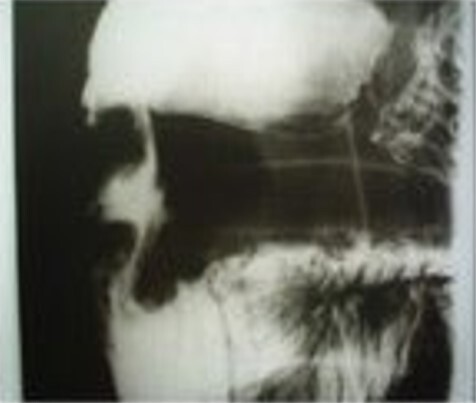
Contrast radiographs showing stenosis of the second portion of the duodenum in a patient with groove pancreatitis who underwent emergency gastroenteroanastomosis.

 EUS-guided biopsy was performed in five patients, revealing chronic inflammatory changes suggestive of chronic pancreatitis in three, while the results were inconclusive in two. Two patients underwent contrast studies of the esophagus, stomach, and duodenum (upper gastrointestinal [UGI] series), which demonstrated stenosis of the second portion of the duodenum (Figure 2). Only two patients showed laboratory evidence of impaired hepatic excretion, suggestive of cholestasis. The preoperative diagnosis of GP was established in five patients, while in three, surgery was indicated due to a suspicion of neoplasia. None of the patients with a preoperative diagnosis of GP had undergone endoscopic treatment, and all were on opioid analgesics with only partial pain relief. Two patients had previously undergone emergency gastroenteroanastomosis due to duodenal obstruction. 

**Figure 3:**
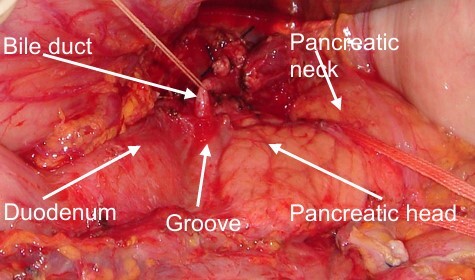
Intraoperative view of a patient undergoing duodenopancreatectomy for groove pancreatitis. The macroscopic features of the pancreatic body appear normal.

 In seven patients, the surgical procedure performed was pylorus-preserving pancreatoduodenectomy (Figures 3 [Fig F5]-5), with a single-loop duct-to-mucosa pancreatojejunostomy. In one patient, the classic Whipple procedure (gastroduodenopancreatectomy) was performed. One patient developed a Grade B pancreatic fistula (12.5%). 

**Figure 4 F5:**
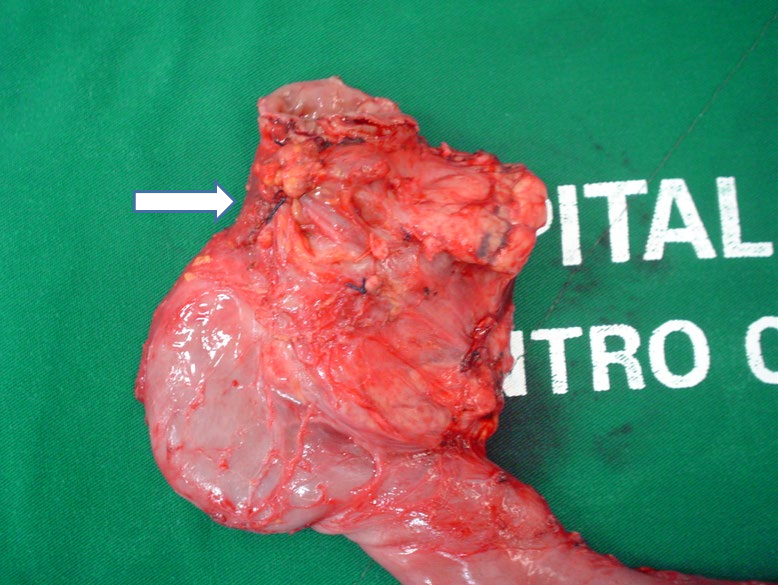
Surgical specimen showing duodenal retraction (arrow).

**Figure 5:**
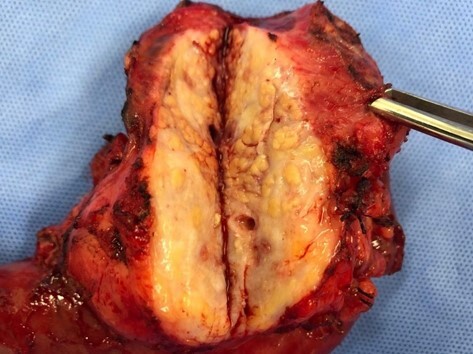
Surgical specimen, section of the groove (pseudotumor), showing the presence of small cysts.

 During long-term follow-up (a mean of 3.5 years), complete pain regression was observed in six patients (75%), while two experienced partial improvement, requiring intermittent use of analgesics. All patients gained weight (Table 1). 

**Table 1:** Analyzed variables of patients with groove pancreatitis.

Patient	1	2	3	4	5	6	7	8
Age	50	48	42	51	57	39	35	45
Gender	M	M	F	M	M	M	M	F
Symptoms	Pain, WL, DGE	Pain, WL, DGE	Pain, WL, DGE	Pain, WL	Pain, WL	Pain, WL, DGE	Pain, WL	Pain, WL
Alcohol use	Yes	Yes	Yes	Yes	Yes	Yes	Yes	Yes
Smoking	Yes	Yes	No	Yes	Yes	Yes	Yes	Yes
Jaundice	No	No	Yes	No	Yes	No	No	No
Imaging	US, CT, EED, MRI	US, CT	US, CT	US, CT	CT, MRI	CT, MRI	CT, MRI	CT, MRI
EUS findings	Yes/tumor	Yes/cyst/GP	Yes/GP	Yes/Inc	No	Yes/cyst/GP	No	Yes/cyst
Preoperative diagnosis	Neoplasia	GP	GP	Neoplasia	Neoplasia	GP/inflammatory	GP	GP
Surgery	PPPD	PPPD	PPPD	PPPD	PPPD	PPPD	PPPD	PD
Complications	No	No	No	No	No	Type B fistula	No	No
HP	GP	GP	GP	GP	GP	GP	GP	GP
Postoperative symptoms	Pain relief, ↑ weight	No pain, ↑ weight	Pain relief, ↑ weight	Pain relief, ↑ weight	Pain relief, ↑ weight	Pain relief, ↑ weight	Pain relief, ↑ weight	Pain relief, ↑ weight
Follow-up	3 years	5 years	7 years	3 years	3 years	2 years	4 years	1 year

M: male; F: female; WL: weight loss; DGE: delayed gastric emptying; US: ultrasound; CT: computed tomography; EED: esophagus, stomach and duodenum x-rayx; MRI: magnetic resonance imaging; EUS: endoscopic ultrasound; GP: groove pancreatitis; Inc: inconclusive; PPPD: pylorus-preserving pancreatoduodenectomy; PD: pancreatoduodenectomy; ↑ weight: weight gain; HP: histopathology.

## DISCUSSION

 GP is a rare, segmental form of chronic pancreatitis that mainly affects individuals between 40 and 50 years of age. It has been referred to by various names in the literature, such as paraduodenal pancreatitis, cystic dystrophy of heterotopic pancreas, duodenal dystrophy, duodenal pancreatic hamartoma, paraduodenal wall cyst, and myoadenomatosis^1^. Its true incidence, as well as its pathophysiological aspects, remains unknown. 

 A retrospective study analyzing the pancreas of 600 patients who underwent surgical treatment for chronic pancreatitis found some degree of involvement of the pancreaticoduodenal groove in 19.5% of cases, thereby proposing the classification of GP into three types: pure, segmental, and diffuse, with the latter corresponding to the classic form of chronic pancreatitis, affecting the body and tail of the pancreas in addition to the pancreaticoduodenal groove^6^. However, most studies limit the classification of GP to the pure and segmental forms. The literature shows that GP primarily affects young adult males. In our patients, the mean age was 45 years, with a male predominance. 

 Although GP is often associated with the presence of ectopic pancreatic tissue in the duodenal wall, which would lead to obstruction of the minor duodenal papilla, a mechanism similar to that produced by hyperplasia of Brunner’s glands and increased viscosity of pancreatic juice triggered by chronic alcohol consumption, these pathophysiological aspects, frequently cited in other studies^1,14,21,23^, were not analyzed in the surgical specimens of our patients. 

 However, similar to the classic form of chronic pancreatitis, GP has also been primarily associated with chronic alcohol consumption and smoking^11,13,19,25^, a finding observed in our sample, in which all patients were heavy alcohol users and only one was not a chronic smoker. 

 The clinical presentation of patients with GP is mainly characterized by progressive abdominal pain of moderate-tosevere intensity, located in the upper abdomen and sometimes radiating to the back^13,25^. In the early stages of the disease, the pain usually responds to common analgesics, while in advanced stages, as seen in most patients in this series, the response is only partial, requiring the use of stronger analgesics such as morphine derivatives. Other frequently observed symptoms include dyspeptic complaints, nausea, and vomiting. 

 Acute gastric emptying disorder due to duodenal obstruction may rarely occur^27^. A total of four patients in our series presented with symptoms consistent with DGE; in two of them, due to symptom severity, urgent gastroenteroanastomosis was required. Pain, dyspeptic symptoms, and malabsorption are the main contributors to the WL commonly observed in these patients. Jaundice due to compression of the main bile duct or fibrosis of pancreatic tissue is rarely seen^17,19^. Only two of our patients had clinical and laboratory evidence of cholestasis at the time of diagnosis. Acute pancreatitis may be the initial manifestation of GP^10,15,25^. Since the earliest reports, one of the main concerns has been differentiating GP from a pancreatic head neoplasm. In approximately 5% of pancreatic resections performed for suspected neoplasia, the diagnosis is ultimately ruled out based on the surgical specimen^14^. 

 Advancements in imaging methods, especially CT and MRI, have significantly improved the diagnosis of GP, allowing for its distinction from pancreatic head neoplasia with high accuracy^13,18,19,25^. However, in some situations, the differential diagnosis remains challenging^22^. In our series, both imaging modalities contributed to the diagnosis of GP in five patients. Similarly, EUS, in addition to describing the characteristic signs of chronic pancreatitis and alterations in the duodenal wall and pancreaticoduodenal groove, allows for targeted biopsy^4,13,25^. 

 In our series, EUS was performed in six patients, confirming the diagnosis of GP in four of them. However, biopsy was performed in five patients but was inconclusive in three. 

 The characteristics described on CT and MRI such as widening of the pancreaticoduodenal groove, thickening of the duodenal wall, and cystic formations in this region, especially in the pure form of GP, allow for differentiation from pancreatic head adenocarcinoma^26^. In cases where pancreatic adenocarcinoma arises in the groove region (groove pancreatic carcinoma), radiologic distinction becomes more difficult^12,16^. Similarly, in the segmental form of GP, this differentiation is not always possible. However, mesenteric vessels are usually preserved in GP, unlike in pancreatic ductal adenocarcinoma^2^. 

 The treatment of GP is initially clinical, similar to the traditional form of chronic pancreatitis, involving the gradual use of analgesics, cessation of alcohol and tobacco consumption, and oral pancreatic enzyme replacement therapy^7^. 

 Therapeutic endoscopy can also be used in cases of obstructive jaundice, with the placement of endoprostheses, duodenal dilation, pseudocyst drainage, or the performance of bypass procedures^13,19,25^. Some case series have reported clinical remission rates of up to 80% in patients treated with a combination of both methods^3^. A recent systematic review reported that conservative and endoscopic treatment achieves a favorable response in approximately 50% of patients^13^. 

 In the face of the failure of these approaches, or in situations where pancreatic cancer could not be entirely ruled out, surgery should be performed. In our case series, all patients were referred from other healthcare facilities with a diagnosis of a pancreatic head neoplasm and were already using opioid analgesics; however, in none of them had the diagnosis of GP been confirmed. 

 The most frequently indicated surgical procedure in cases of GP unresponsive to conservative treatment or in the presence of diagnostic uncertainty was, in the vast majority of cases, PD^8,9,11,13,24,25^. Duodenum-preserving pancreatic head resections, as well as biliary and gastric bypass procedures, have also been reported in patients with confirmed GP who were not clinically fit for pancreatic head resection^19^. 

 The results of PD in different published case series show clinical success rates exceeding 80% in terms of pain control and improvement in nutritional status. However, this complex procedure is not free from complications and should preferably be performed in referral centers. A recent systematic review analyzing the various treatment modalities demonstrates that surgical treatment is indeed the most effective approach for controlling the symptoms of this disease^11,13,25^. 

## CONCLUSIONS

 GP is a rare, segmental form of chronic pancreatitis that, in some situations, may be mistaken for pancreatic head neoplasia, with imaging methods and EUS currently playing an important role in its diagnosis. A review of the medical papers shows that initial treatment should be multidisciplinary, similar to the approach used for classic chronic pancreatitis. When there is little or no response to conservative and/or endoscopic treatment, surgical intervention is indicated, and PD is an excellent option, provided it is performed in hospitals with extensive experience in pancreatobiliary surgery. 

## Data Availability

The information regarding the investigation, methodology, and data analysis of the article is archived under the responsibility of the authors.

## References

[1] Adsay NV, Zamboni G (2004). Paraduodenal pancreatitis: a clinico-pathologically distinct entity unifying "cystic dystrophy of heterotopic pancreas", "para-duodenal wall cyst", and "groove pancreatitis". Semin Diagn Pathol.

[2] Addeo G, Beccani D, Cozzi D, Ferrari R, Lanzetta MM, Paolantonio P (2019). Groove pancreatitis: a challenging imaging diagnosis. Gland Surg.

[3] Arvanitakis M, Rigaux J, Toussaint E, Eisendrath P, Bali MA, Matos C (2014). Endotherapy for paraduodenal pancreatitis: a large retrospective case series. Endoscopy.

[4] Ashkar M, Gardner TB (2014). Role of endoscopic ultrasound in pancreatic diseases: a systematic review. Minerva Gastroenterol Dietol.

[5] Becker V (1973). Proceedings: Fundamental morphological aspects of acute and chronic pancreatitis (author’s transl). Langenbecks Arch Chir.

[6] Becker V, Mischke U (1991). Groove pancreatitis. Int J Pancreatol.

[7] Beyer G, Habtezion A, Werner J, Lerch MM, Mayerle J (2020). Chronic pancreatitis. Lancet.

[8] Casetti L, Bassi C, Salvia R, Butturini G, Graziani R, Falconi M (2009). "Paraduodenal" pancreatitis: results of surgery on 58 consecutives patients from a single institution. World J Surg.

[9] Dhali A, Ray S, Ghosh R, Misra D, Dhali GK (2022). Outcome of Whipple’s procedure for Groove pancreatitis: a retrospective cross-sectional study. Ann Med Surg (Lond).

[10] Değer KC, Köker İH, Destek S, Toprak H, Yapalak O, Gönültaş C (2022). The clinical feature and outcome of groove pancreatitis in a cohort: a single center experience with review of the literature. Ulus Travma Acil Cerrahi Derg.

[11] Egorov V, Petrov R, Schegolev A, Dubova E, Vankovich A, Kondratyev E (2021). Pancreas-preserving duodenal resections vs pancreatoduodenectomy for groove pancreatitis. Should we revisit treatment algorithm for groove pancreatitis?. World J Gastrointest Surg.

[12] Gabata T, Kadoya M, Terayama N, Sanada J, Kobayashi S, Matsui C (2003). Groove pancreatic carcinomas: radiological and pathological findings. Eur Radiol.

[13] Kager LM, Lekkerkerker SJ, Arvanitakis M, Delhaye M, Fockens P, Boermeester MA (2017). Outcomes after conservative, endoscopic, and surgical treatment of groove pancreatitis: a systematic review. J Clin Gastroenterol.

[14] Levenick JM, Gordon SR, Sutton JE, Suriawinata A, Gardner TB (2009). A comprehensive, case-based review of groove pancreatitis. Pancreas.

[15] Li J, Liu Q, Liu Z, Cen C, Yang Y, Ye J (2021). Acute pancreatitis associated with duodenal obstruction induced by groove pancreatitis: a case report. Medicine (Baltimore).

[16] Miller FH, Vendrami CL, Hammond NA, Mittal PK, Nikolaidis P, Jawahar A (2023). Pancreatic cancer and its mimics. Radiographics.

[17] Nascimento CN, Palmela C, Soares AS, Antunes ML, Fidalgo CA, Glória L (2022). Groove pancreatitis: clinical cases and review of the literature. GE Port J Gastroenterol.

[18] Oza VM, Skeans JM, Muscarella P, Walker JP, Sklaw BC, Cronley KM (2015). Groove pancreatitis, a masquerading yet distinct clinicopathological entity: analysis of risk factors and differentiation. Pancreas.

[19] Pallisera-Lloveras A, Ramia-Ángel JM, Vicens-Arbona C, Cifuentes-Rodenas A (2015). Groove pancreatitis. Rev Esp Enferm Dig.

[20] Silva EBS, Silva MC, Araújo MCS, Paulino BMSL, Moraes JMA, Torres OJM (2024). Pancreatoduodenectomy as treatment for recurrent acute pancreatitis due to pancreas divisum. Arq Bras Cir Dig.

[21] Stolte M, Weiss W, Volkholz H, Rösch W (1982). A special form of segmental pancreatitis: "groove pancreatitis". Hepatogastroenterology.

[22] Tan CH, Chow PKH, Thng CH, Chung AYF, Wong WK (2006). Pancreatic adenocarcinoma that mimics groove pancreatitis: case report of a diagnostic dilemma. Dig Dis Sci.

[23] Tezuka K, Makino T, Hirai I, Kimura W (2010). Groove pancreatitis. Dig Surg.

[24] Torres OJM, Vasques RR, Barros CM, Sauaia GA, Mouchrek BDM (2023). Pancreatoduodenectomy due to lipomatous pseudohypertrophy of the pancreas. Arq Bras Cir Dig.

[25] Ukegjini K, Steffen T, Tarantino I, Jonas JP, Rössler F, Petrowsky H (2023). Systematic review on groove pancreatitis: management of a rare disease. BJS Open.

[26] Zaheer A, Haider M, Kawamoto S, Hruban RH, Fishman EK (2014). Dual-phase CT findings of groove pancreatitis. Eur J Radiol.

[27] Zhang Y, Cheng HH, Fan WJ (2023). Duodenojejunostomy treatment of groove pancreatitis-induced stenosis and obstruction of the horizontal duodenum: a case report. World J Gastrointest Surg.

